# Allogeneic ADSCs induce CD8 T cell-mediated cytotoxicity and faster cell death after exposure to xenogeneic serum or proinflammatory cytokines

**DOI:** 10.1038/s12276-019-0231-5

**Published:** 2019-03-11

**Authors:** Sung-Ho Chang, Chung-Gyu Park

**Affiliations:** 1 Institute of Endemic Diseases, Medical Research Center, Seoul National University College of Medicine, Seoul, Republic of Korea; 2 Xenotransplantation Research Center, Seoul National University College of Medicine, Seoul, Republic of Korea; 3 Department of Microbiology and Immunology, Seoul National University College of Medicine, Seoul, Republic of Korea; 4 Department of Biochemistry and Molecular Biology, Asan Medical Center, University of Ulsan College of Medicine, Seoul, Republic of Korea; 5 Department of Biomedical Sciences, Seoul National University College of Medicine, Seoul, Republic of Korea; 6 Cancer Research Institute, Seoul National University College of Medicine, Seoul, Republic of Korea

**Keywords:** Mesenchymal stem cells, Allotransplantation, Lymphocyte activation

## Abstract

This study examined the induction of recipient T-cell cytotoxicity after exposure to allogeneic adipose-derived mesenchymal stem cells (ADSCs). ADSCs pre-exposed to xenogeneic serum significantly induced cytotoxicity through CD8 T-cell granzyme B secretion after allogeneic antigen stimulation, and this effect was increased with prolonged reaction time. ADSCs pretreated with proinflammatory cytokines also induced cytotoxicity through granzyme B secretion and significantly increased human leukocyte antigen (HLA)-ABC expression. T-cell cytotoxicity towards ADSCs grown in xeno-free medium (XF-ADSCs) was lower than that towards ADSCs exposed to xenogeneic serum or proinflammatory cytokines, but XF-ADSCs still induced cytotoxicity. We further investigated the causes of T-cell cytotoxicity towards XF-ADSCs. XF-ADSC death was effectively inhibited by HLA-blocking antibodies, suggesting that ADSC HLAs are a major cause of alloreactive T-cell generation. These results indicated that culturing of allogeneic ADSCs with recipient serum may alleviate alloreactive CD8 T-cell cytotoxicity. Ultimately, development of therapeutic agents using autologous ADSCs would be a suitable way to avoid immunogenicity and CD8 T cell-mediated cytotoxicity, but more attention should be paid to the potential immunogenicity of allogeneic ADSCs, which could perhaps be mitigated through the use of immunosuppressants.

## Stem cell therapy: Limiting immune response attacks

The conditions under which donor stem cells are cultured can limit attack by the recipient’s immune system after transplantation. Adult stem cells taken from donors who are genetically similar to recipients show promise as therapeutic agents for various conditions, from cardiac to immunity-related diseases. However, patients’ immune systems will often attack and destroy the transplanted cells. Chung-Gyu Park and Sung-Ho Chang at Seoul National University, South Korea, explored methods of growing stem cells so that they are less likely to be destroyed by the patient’s T-cells. The team found that human T-cells will attack stem cells grown in media containing bovine serum or those pre-treated with pro-inflammatory proteins. T-cell activity was weaker against stem cells grown in media with autologous serum.

## Introduction

Human mesenchymal stem cells (MSCs) proliferate and differentiate in response to signals in their surrounding environment and display immunomodulating, angiogenic, and self-renewing abilities. Therefore, they have attracted attention as potential therapeutic agents for cardiac, neurological, orthopedic, digestive, and immune diseases^1–4^. In contrast to embryonic stem cells, MSCs do not develop teratomas and are relatively safe after implantation; thus, they are widely used in the development of therapeutic agents^4,5^. In the early stages, MSC treatments were developed using mostly autologous cells to minimize the immune response, but the use of allogeneic cells, which can be mass produced, is gradually increasing^6,7^.

MSCs do not express major histocompatibility complex (MHC) class II molecules or costimulatory molecules such as CD40, CD80, and CD86, and they have low expression of MHC class I molecules^8,9^. Therefore, MSCs are thought to possess no or low immunogenicity in allografts^10–12^. In addition, MSCs exhibit immunomodulatory activity and, clinically, therapeutic effects against immunological diseases can be expected^13,14^. However, there is a concern that allogeneic MSCs may be immunogenic due to the expression of allogeneic antigens at the allograft^15–24^. In addition, MSCs do not have immunosuppressive effects when applied to animal models of immunological disease; rather, they can exacerbate the disease^25^.

T cells can initiate an immune response through recognition of specific antigens in allograft donor cells. The antigens on the surface of the donor cell are called MHC molecules, and the recipient T cell can recognize the intact MHC molecules or the donor MHC peptides bound to the MHC molecules of the recipient antigen-presenting cell (APC). In the traditional model, CD4 T cells can recognize MHC class II molecules, and CD8 T cells can recognize MHC class I molecules. CD8 T cells can differentiate into cytotoxic T lymphocytes (CTLs) produced by direct allorecognition and can kill donor cells^22,26^. CTLs contribute to the death of target cells in different ways, such as through apoptosis and necrosis^27–30^.

To use allogeneic MSCs clinically, it is important to be able to predict their immunogenicity prior to administration to the patient, as an immune response after administration may result in decreased cell viability and therapeutic efficacy. Thus, predicting changes in immunogenicity in response to different conditions of MSC exposure will be important for achieving the clinical objective of allogeneic MSC use^31,32^.

In this study, we investigated the effects of allogeneic adipose-derived mesenchymal stem cells (ADSCs) previously exposed to xenogeneic serum or proinflammatory cytokines on the cytotoxicity of the recipient immune system. In addition, the generation and cause of the effect of alloreactive T cells on XF-ADSCs were investigated. Cytotoxicity was assessed through analysis of ADSC viability and death. Thus, this study aimed to identify the optimal conditions for ADSC transplantation and determine the immunogenicity of ADSCs through cytotoxicity experiments.

## Materials and methods

### Preparation of human ADSCs

Human ADSCs were isolated from abdominal or breast adipose tissue, treated with collagenase type I (Life Technologies, Grand Island, NY, USA), and then cultured in xeno-free medium (CellGenix, Portsmouth, NH, USA, 24803-0500) without animal-derived components for 1 day in a T-75 flask (Thermo Fisher, Carlsbad, CA, USA) coated with CELLstart humanized substrate (Life Technologies, A1014201)^33^. Floating cells were removed the next day by replacing the medium. Verification of isolated ADSCs was performed using antibodies against CD44, CD105, CD73, and CD90 (eBioscience, San Diego, CA, USA). The isolated ADSCs did not express CD80, CD86, or human leukocyte antigen (HLA)-DR. To screen the ADSC surface antigens, the cells were analyzed using antibodies against HLA-ABC and corresponding isotypes (eBioscience). Surface type analysis of the ADSCs was performed using a FACSCanto II flow cytometer (BD Biosciences, San Diego, CA, USA).

### Preparation of T cells and Td-PBMCs for allogeneic antigen stimulation

For allogeneic antigen stimulation, peripheral blood mononuclear cells (PBMCs) were isolated from blood donated by healthy people, and CD3, CD4, and CD8 T cells were isolated using specific kits (Miltenyi Biotec, Auburn, CA, USA) according to the manufacturer’s protocols. To measure the activity of antigen-specific T cells, the cells were labeled with 0.5 μM 5(6)-carboxyfluorescein diacetate *N*-succinimidyl ester (CFSE; Sigma-Aldrich, St Louis, MO, USA) for 5 min at room temperature and then washed four times with Dulbecco’s phosphate-buffered saline (DPBS). T cell-depleted PBMCs (Td-PBMCs) were isolated by removing T cells using a CD3 T-cell isolation kit (Miltenyi Biotec). Td-PBMCs were used as APCs through the B cells contained within these cells and were irradiated at 10 Gy because of a reported decrease in APC function at 20 Gy^33–35^. T cells with low CFSE staining (CFSE-low T cells) were analyzed by flow cytometry, and the data were analyzed using FlowJo version 7.6.5 software (Ashland, OR, USA).

### Allogeneic antigen stimulation

Isolated ADSCs cultured in xeno-free medium are hereafter referred to as XF-ADSCs in this study. For exposure to xenogeneic serum, ADSCs were cultured in Dulbecco’s modified Eagle’s medium (DMEM) supplemented with 10% fetal bovine serum (FBS), 1% penicillin–streptomycin, and 1% GlutaMAX (Life Technologies), and then the medium was exchanged with xeno-free medium 3 or 1 days prior to allogeneic antigen stimulation; these cells are referred to as −3d XF-ADSCs or −1d XF-ADSCs, respectively. For immunogenicity studies on surface antigen-altered ADSCs, the cells were stimulated with a combination of interferon-γ (IFN-γ; 50 ng/ml, 34-8319-85), interleukin (IL)-17A/F (50 ng/ml, 34-8178-85), and IL-23 (10 ng/ml, 14-8239-63) for 3 days (eBioscience). For the direct pathway, prepared ADSCs were seeded at a density of 1 × 10^3^ cells/well in a 12-well plate on the day of allogeneic antigen stimulation. CD3 T, CD4 T, or CD8 T cells were seeded at densities of 1.8 × 10^5^, 1.2 × 10^5^, and 6 × 10^4^ cells/well, respectively, and Td-PBMCs were seeded at a density of 1 × 10^5^ cells/well. Td-PBMCs were further added on days 7 and 14 for allogeneic antigen stimulation and on day 14 for enzyme-linked immunospot (ELISPOT) reactions. ADSCs (1 × 10^3^ cells/well) were added on day 7 for allogeneic antigen stimulation. For the indirect pathway, ADSCs were disrupted by repetitive thawing and freezing (5 times) using liquid nitrogen, and then the disrupted ADSCs were seeded at a density of 1 × 10^3^ cells/well in a 12-well plate for analysis of the antigen recognition pathway. The medium for allogeneic antigen stimulation was composed of xeno-free medium with 5% autologous serum from a blood donor, 1 ng/ml IL-2 (eBioscience), 1% penicillin–streptomycin, and 1% GlutaMAX™. Half of the culture was replaced with fresh medium every 7 days.

### ELISPOT analysis

ELISPOT was performed to measure granzyme B and IFN-γ secretion from T cells after allogeneic antigen stimulation. An anti-human granzyme B capture antibody (Mabtech, West Street, OH, USA, 3485-2 A) and an anti-human IFN-γ capture antibody (BD Biosciences) were coated overnight at 4 °C onto 96-well filter plates (Millipore, Billerica, MA, USA, S2EM004M99) according to the manufacturer’s instructions. At 14 days after allogeneic antigen stimulation, all cells from each well of the 12-well plates were collected, washed with DPBS, and seeded in quadruplicate in the antibody-coated 96-well filter plates. After 3 days of additional allogeneic antigen stimulation, the cells were removed, a biotin-conjugated detection antibody was added, and a streptavidin-alkaline phosphatase conjugate was added according to the manufacturer’s instructions (Mabtech). The plate was developed using nitro-blue tetrazolium and 5-bromo-4-chloro-3’-indolylphosphate (Sigma-Aldrich), and the spots were analyzed on an ELISPOT reader (AID GmbH, Strassberg, Germany).

### ADSC viability analysis

ADSC viability was analyzed using a Cell Counting Kit-8 (CCK-8; Dojindo Molecular Technologies, Kumamoto, Japan, CK04-11) after 7 or 14 days of allogeneic antigen stimulation. For this analysis, the floating cells were carefully removed from the mixed cells by rinsing the plates 1–2 times with warm DPBS at 37 °C. DMEM (0.3 ml) was added to each well, and then 30 μl of CCK-8 was added; the mixture was then incubated in a CO_2_ incubator for 3–4 h. Portions (100 μl) of each sample were transferred to a 96-well plate, and the cell viability was evaluated by analyzing the absorbance at 450 nm on a Multiskan Ascent plate reader (Thermo Labsystems, Helsinki, Finland).

### ADSC death analysis

ADSC death was analyzed 20–24 days after allogeneic antigen stimulation. For this analysis, the plates were rinsed carefully with warm DPBS at 37 °C to remove floating cells. Attached ADSCs were isolated using Accutase, and the isolated ADSCs were stained with antibodies against the ADSC markers CD73 and CD90 (BioLegend, San Diego, CA, USA). To analyze the ADSC apoptosis and necrosis ratios, the cells were reacted with annexin V in annexin V binding buffer and then stained with propidium iodide (PI; eBioscience). The stained ADSCs were analyzed by flow cytometry.

### Antibody treatment

Neutralizing antibodies against proinflammatory cytokines (anti-human IFN-γ (eBioscience, 14-7318-85) and anti-human IL-17A (eBioscience, 16-7178-85)) were used during allogeneic antigen stimulation. Anti-HLA-ABC (BioLegend, 311412), anti-HLA-DR (BioLegend, 307612), and anti-HLA-DQ (BioLegend, 361502) antibodies were used to block ADSC surface antigens. All of these antibodies were added at a concentration of 1 μg/ml every 7 days when exchanging the medium.

### Statistics

Student’s *t*-test was used for comparisons between two experimental groups. One-way analysis of variance with Bonferroni’s test was used for multiple comparisons. All analyses were performed using GraphPad Prism, version 4 (GraphPad Software Inc., San Diego, CA, USA). All data are expressed as the mean ± SEM, and asterisks indicate significant differences from the control group (*p < 0.05; ***p < 0.001). All experiments were repeated at least two times.

### Study approval

Human adipose tissue, obtained with patient consent, was provided by Professors I.H. Oh and J.W. Rhie in accordance with procedures approved by the Catholic University in Seoul, Korea. The Institutional Review Board of the Hospital Biomedical Research Institute of Seoul National University approved this study (document number: 1403-036-563).

## Results

### Allogeneic ADSCs previously exposed to xenogeneic serum induce greater CD8 T cell-mediated cytotoxicity than XF-ADSCs

**Fig. 1:**
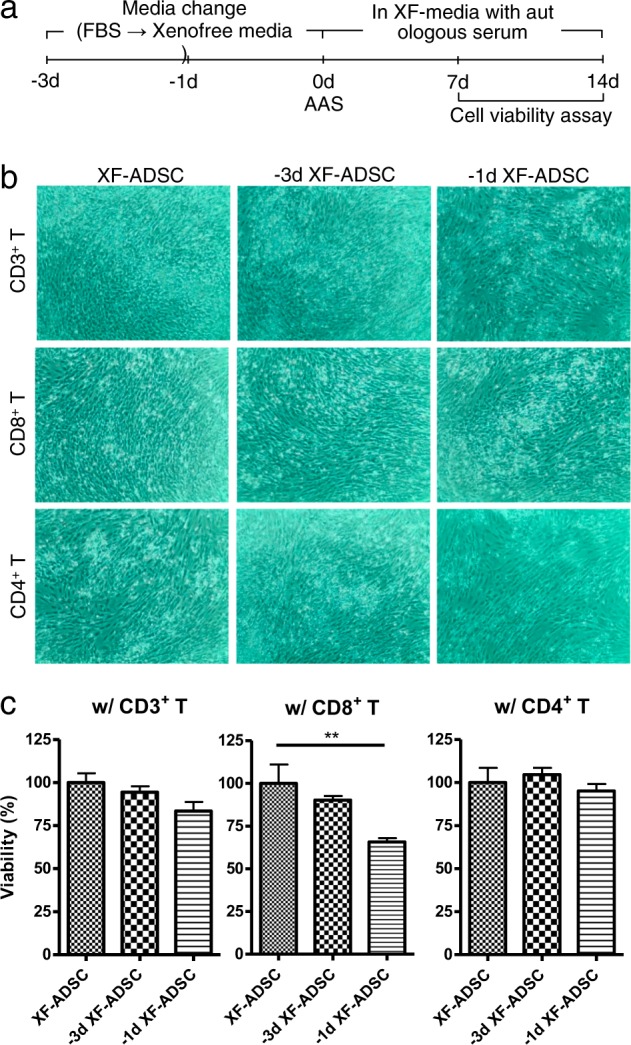
Adipose-derived mesenchymal stem cells (ADSCs) previously exposed to xenogeneic serum induce cytotoxicity in recipient CD8 T cells. Human ADSCs were subjected to allogeneic antigen stimulation for 7 days, and then ADSC viability was analyzed. **a** The 7- and 14-day experimental schemes. **b** For allogeneic antigen stimulation, T cells and T cell-depleted peripheral blood mononuclear cells (Td-PBMCs) were incubated for 7 days with allogeneic ADSCs in xeno-free medium (XF-ADSCs) containing 5% autologous serum. XF-ADSCs were cultured in xeno-free medium immediately after isolation, and −3d XF-ADSCs or −1d XF-ADSCs were changed from fetal bovine serum (FBS)-containing medium to xeno-free medium 3 days or 1 day before the experiment, respectively. XF-ADSCs were used as controls for −3d XF-ADSCs and −1d XF-ADSCs in the cell viability assay. Human CD4, CD8, and CD3 T cells were used as recipient T cells. **c** Graphical representation of ADSC viability corresponding to (**b**) (*n* = 6 for each sample); **p < 0.001. AAS allogeneic antigen stimulation

Human ADSCs can be cultured under various conditions, depending on the research purpose or preferred culture method of the individual laboratory. For proliferation, for example, ADSCs are cultured in medium containing xenogeneic serum. This study examined the effects of these culture conditions on the immunogenicity of allogeneic ADSCs. We used ADSCs cultured under the following two conditions for allogeneic antigen stimulation in an ex vivo immunogenicity assessment model: (1) ADSCs cultured in xeno-free medium immediately after isolation from adipose tissue (XF-ADSCs), and (2) ADSCs cultured in DMEM containing FBS that were washed with DPBS 1 or 3 days before allogeneic antigen stimulation, at which time the medium was replaced with xeno-free medium. The viability of these ADSCs was tested 7 days after allogeneic antigen stimulation with recipient immune cells composed of T cells and Td-PBMCs in xeno-free medium containing autologous serum. As shown in Fig. 1, the viability of −1d XF-ADSCs cultured with CD8 T cells was significantly lower than that of XF-ADSCs. However, when cultured with CD4 T cells, none of the ADSCs displayed decreased viability compared with XF-ADSCs. These results suggest that CD8 T cells may exhibit greater cytotoxicity against −1d XF-ADSCs, which had been previously exposed to xenogeneic serum, than against XF-ADSCs.

### The decreased viability of pre-exposed allogeneic ADSCs is associated with an increase in CD8 T-cell granzyme B production, and the cytotoxic effect was reaction time dependent

**Fig. 2:**
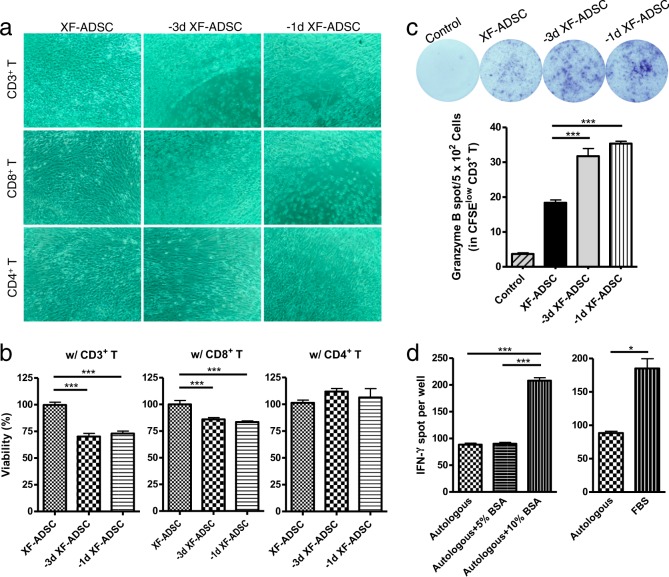
Pre-exposed adipose-derived mesenchymal stem cells (ADSCs) increase cytotoxicity through CD8 T-cell granzyme B production with increasing reaction times. **a** ADSCs previously exposed to xenogeneic serum were subjected to allogeneic antigen stimulation for 14 days in xeno-free medium containing 5% autologous serum. Pre-exposed ADSCs were reacted with each T cell type, and the cell viability was evaluated in comparison with that of ADSCs grown in xeno-free medium (XF-ADSCs), which were cultured in xeno-free medium immediately after isolation from adipose tissue. **b** Graphical representation of ADSC viability corresponding to (**a**) (*n* = 12 (each for CD3 T and CD8 T), *n* = 6 (CD4 T)). **c** Spots of granzyme B secreted by CD3 T cells were analyzed by enzyme-linked immunospot (ELISPOT) analysis (*n* = 3 for each sample). **d** CD3 T cells and T cell-depleted peripheral blood mononuclear cells (Td-PBMCs) were cultured in medium containing 5% autologous serum, bovine serum albumin (BSA) or fetal bovine serum (FBS) for 21 days. To examine the effect of xenogeneic antigens, the production of interferon-γ (IFN-γ) by T cells was analyzed by ELISPOT (*n* = 6 (autologous), *n* = 3 (each for BSA and FBS)); *p < 0.05; ***p < 0.001

To further investigate the cytotoxic effects of recipient T cells on allogeneic ADSCs previously exposed to xenogeneic serum, the duration of allogeneic antigen stimulation was extended. Pre-exposed ADSCs were subjected to allogeneic antigen stimulation with CD3, CD8, or CD4 T cells for 14 days, followed by evaluation of ADSC viability. Both −1d XF-ADSCs and −3d XF-ADSCs showed markedly lower viability in the presence of CD3 T cells than did XF-ADSCs (Fig. 2). Similarly, the viability of ADSCs in the presence of CD8 T cells was also significantly lower than that of XF-ADSCs. However, even after 14 days, pre-exposed ADSCs cultured with CD4 T cells did not display lower cell viability than XF-ADSCs. We further investigated the mechanism of T-cell cytotoxicity towards ADSCs previously exposed to xenogeneic serum by analyzing the production of granzyme B from CFSE-low T cells by ELISPOT 14 days after allogeneic antigen stimulation. As shown in Fig. 2c, CFSE-low CD3 T cells produced significantly more granzyme B in response to −1d XF-ADSCs and −3d XF-ADSCs than in response to XF-ADSCs or control solution not containing XF-ADSCs. These results suggest that the cytotoxicity of T cells towards ADSCs previously exposed to xenogeneic serum was significantly higher than that against XF-ADSCs and that this result was induced by granzyme B secreted from CD8, but not CD4, T cells (Fig. 2a–c). This phenomenon occurred not only in CD8 T cells but also in CD3 T cells as the reaction time increased. This finding further suggests that XF-ADSCs, which were used as controls for pre-exposed ADSCs, induced granzyme B production by CD3 T cells, suggesting that further immunogenicity testing is needed for XF-ADSCs.

To further investigate the effect of xenogeneic serum on human T cells and Td-PBMCs, these cells were incubated with FBS or bovine serum albumin (BSA) for 14 days, and then T-cell IFN-γ secretion was analyzed by ELISPOT. Human T cells cultured in 10% FBS or 10% BSA showed increased IFN-γ production compared to T cells cultured in autologous serum. These results suggest that culturing T cells with xenogeneic serum further increases the activity of the recipient immune cells compared to culturing with autologous serum.

### Allogeneic XF-ADSCs pretreated with proinflammatory cytokines induce greater CD8 T cell-mediated cytotoxicity than untreated XF-ADSCs

**Fig. 3:**
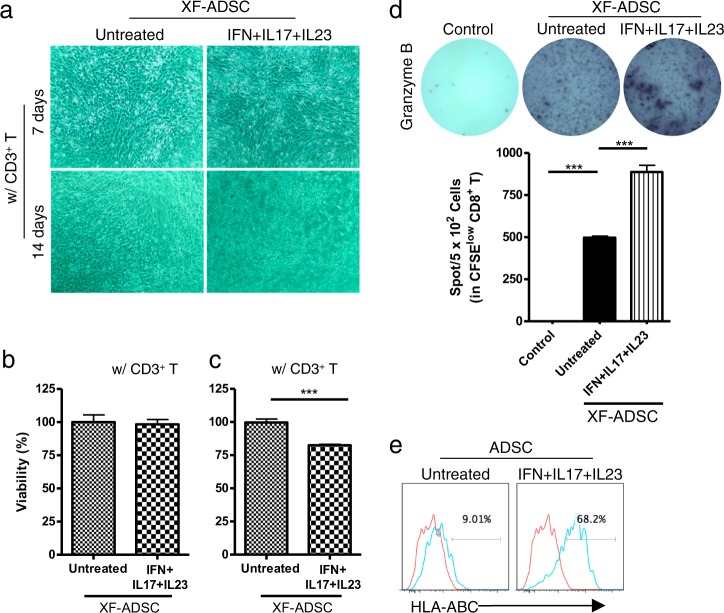
Adipose-derived mesenchymal stem cells grown in xeno-free medium (XF-ADSCs) pretreated with proinflammatory cytokines induced recipient CD8 T cell-mediated cytotoxicity through granzyme B secretion. **a** XF-ADSCs pretreated with a combination of interferon-γ (IFN-γ), interleukin (IL)-17, and IL-23 were subjected to allogeneic antigen stimulation for 7 or 14 days in xeno-free medium containing 5% autologous serum. The pretreated XF-ADSCs were reacted with each T-cell type, and the cell viability was evaluated in comparison with that of untreated XF-ADSCs. **b** Graphical representation of XF-ADSC viability after 7 days corresponding to (**a**) (*n* = 6 for each sample). **c** Graphical representation of XF-ADSC viability after 14 days corresponding to (**a**) (*n* = 12 for each sample). **d** Spots of granzyme B secreted by CD8 T cells were analyzed by enzyme-linked immunospot (ELISPOT) analysis (*n* = 4 for each sample). **e** Representative analysis of human leukocyte antigen (HLA)-ABC expression levels in untreated ADSCs or IFN-γ-, IL-17-, and IL-23-treated ADSCs by flow cytometry; ***p < 0.001

We investigated the effects of the recipient T cells on allogeneic XF-ADSCs pretreated with IFN-γ, IL-17, and IL-23 complexes in xeno-free medium 3 days prior to allogeneic antigen stimulation. The pretreated XF-ADSCs were then incubated with CD3 T cells and Td-PBMCs for allogeneic antigen stimulation. After 7 days of allogeneic antigen stimulation, the viability of the pretreated XF-ADSCs was not decreased compared with that of the untreated XF-ADSCs (Fig. 3). However, 14 days after allogeneic antigen stimulation, CD3 T cells significantly decreased the viability of pretreated XF-ADSCs compared to that of untreated XF-ADSCs.

To examine the cytotoxic mechanism, we measured the amount of granzyme B produced by CFSE-low CD8 T cells. As shown in Fig. 3d, pretreated XF-ADSCs caused significantly higher production of granzyme B by CFSE-low CD8 T cells than did the control solution (with CD3 T and Td-PBMC) not containing XF-ADSCs. In addition, these results demonstrated that the production of granzyme B by CD8 T cells can be induced by XF-ADSCs as well as by pretreated XF-ADSCs.

We next examined the expression of HLA-ABC to understand the relationship between the cytotoxic effects of CD8 T cells and HLA-ABC expression on ADSCs. ADSCs treated with the combination of IFN-γ, IL-17, and IL-23 displayed significantly increased HLA-ABC expression compared to untreated ADSCs (Fig. 3e). These results suggest that a marked increase in HLA-ABC antigen expression by allogeneic ADSCs might be associated with the increased activity of alloreactive CD8 T cells (Fig. 3).

### Allogeneic XF-ADSCs induce T-cell cytotoxicity that is delayed compared with that induced by ADSCs previously exposed to xenogeneic serum or proinflammatory cytokines

**Fig. 4:**
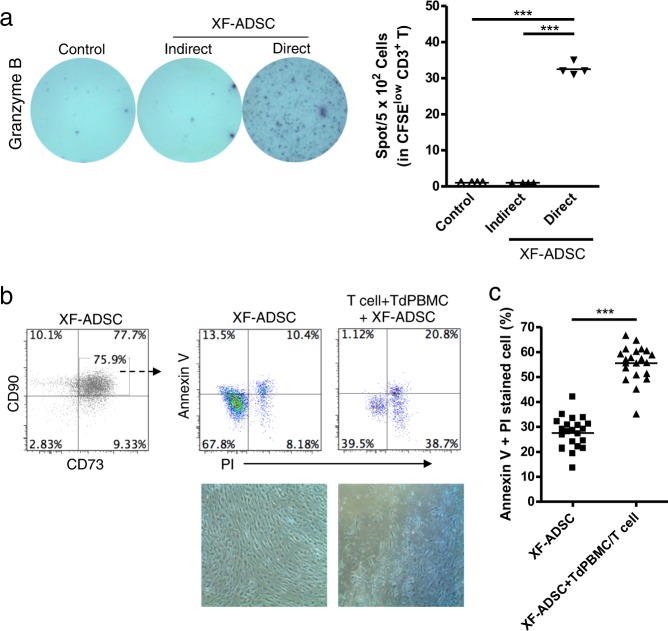
Adipose-derived mesenchymal stem cells grown in xeno-free medium (XF-ADSCs) cause delayed T cell-mediated cytotoxicity through a direct pathway. **a** Comparison of antigen recognition through direct and indirect pathways in allogeneic antigen stimulation, as described for the enzyme-linked immunospot (ELISPOT) analysis in the Materials and methods. Spots of granzyme B secreted by CD3 T cells were analyzed by ELISPOT analysis (*n* = 4 for each sample). **b** XF-ADSCs were subjected to allogeneic antigen stimulation for 21–24 days in xeno-free medium containing 5% autologous serum. For cell death analysis, the collected XF-ADSCs were stained with anti-CD73 and anti-CD90 antibodies, annexin V, and propidium iodide (PI). The rate of XF-ADSC death induced by T cells was compared with that of control XF-ADSCs not containing recipient immune cells after analysis by flow cytometry. **c** Graphical representation of XF-ADSC death corresponding to (**b**) (*n* = 22 for each sample); ***p < 0.001. Td-PBMCs T cell-depleted T cell-depleted peripheral blood mononuclear cells

The above experiments demonstrated that XF-ADSCs could induce granzyme B production by CD8 T cells. We examined the antigen recognition pathway of alloreactive T cells for the production of granzyme B. In allograft rejection, T cells can recognize antigens through direct and indirect pathways, and direct pathways are associated with acute rejection^26^. XF-ADSCs significantly induced the production of granzyme B through the direct pathway rather than the indirect pathway (Fig. 4a). This result implied that granzyme B might be associated with allogeneic rejection of XF-ADSCs. Thus, we investigated whether allogeneic XF-ADSCs could induce cell death via alloreactive T cell-induced cytotoxicity. The induction of apoptosis and necrosis in XF-ADSCs by T cells after allogeneic antigen stimulation was analyzed with annexin V and PI staining and flow cytometry. As shown in Fig. 4b, c, the sum of the apoptosis and necrosis rates of XF-ADSCs was double that of XF-ADSCs not containing recipient immune cells 21–24 days after allogeneic antigen stimulation. These results indicated that even allogeneic XF-ADSCs cultured in xeno-free medium can induce cytotoxicity mediated by recipient T cells, but this occurs approximately 1 week later than that induced by ADSCs pre-exposed to xenogeneic serum or pretreated with proinflammatory cytokines (Figs. 2–4).

### The major cause of cytotoxicity towards allogeneic XF-ADSCs is HLA antigens

**Fig. 5:**
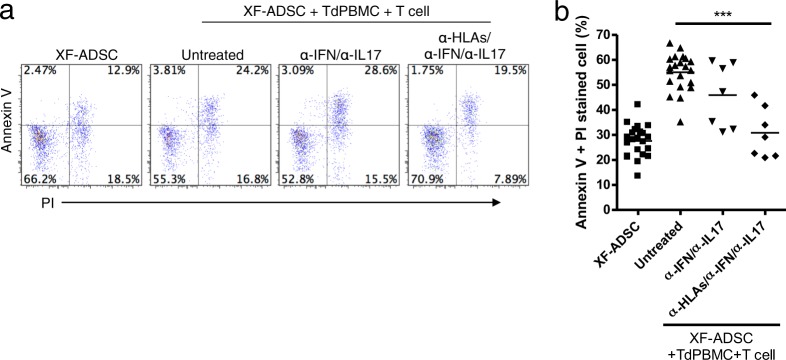
Human leukocyte antigens (HLAs) on allogeneic adipose-derived mesenchymal stem cells grown in xeno-free medium (XF-ADSCs) are the major cause of T cell-mediated cytotoxicity. **a** XF-ADSCs were subjected to allogeneic antigen stimulation for 21–24 days with antibodies against interferon-γ (IFN-γ), interleukin (IL)-17A, HLA-ABC, HLA-DR, and HLA-DQ (all at 1 μg/ml) in xeno-free medium containing 5% autologous serum. The effects of the XF-ADSCs with antibodies on T cell-mediated cytotoxicity were evaluated in comparison with those of the XF-ADSCs not containing antibodies. For cell death analysis, the collected XF-ADSCs were stained with anti-CD73 and anti-CD90 antibodies, annexin V, and propidium iodide (PI), and the cell death rate was analyzed by flow cytometry. **b** Graphical representation of XF-ADSC death corresponding to (**a**) (*n* = 21 (untreated), *n* = 7 (for each sample)); ***p < 0.001. Td-PBMCs T cell-depleted T cell-depleted peripheral blood mononuclear cells

To identify the factors responsible for T-cell cytotoxicity towards allogeneic XF-ADSCs during allogeneic antigen stimulation, we further investigated the relationship between cytotoxic effects and HLA expression on ADSCs. We examined whether neutralizing antibodies corresponding to IL-17A and IFN-γ or blocking antibodies corresponding to HLAs could effectively inhibit T cell-mediated cytotoxicity during allogeneic antigen stimulation. T-cell cytotoxicity towards allogeneic XF-ADSCs was effectively suppressed by anti-HLA antibodies but not by cytokine-neutralizing antibodies (Fig. 5), indicating that the major cause of T cell-mediated cytotoxicity is the expression of HLA antigens on ADSCs.

## Discussion

The purpose of this study was to evaluate whether cytotoxic effects of recipient T cells are induced by allogeneic ADSCs, which are attractive therapeutic agents for regenerative medicine and immunotherapy. In addition, we examined the factors influencing the cytotoxicity of T cells towards allogeneic ADSCs using autologous serum and an ex vivo allogeneic antigen stimulation model to evaluate the immunogenicity of allogeneic ADSCs^36^. To investigate factors that may affect cytotoxicity, we compared the effects of ADSCs exposed to either xenogeneic serum or inflammatory conditions before allogeneic antigen stimulation with those of XF-ADSCs. Previous reports have raised concerns that xenogeneic serum may induce immunogenicity during stem cell transplantation and that proinflammatory cytokines such as IFN-γ may increase immunogenicity by increasing HLA-ABC surface antigens on MSCs^31,32,37^. In addition, Li and Lin^38^ reported that activation of the complement system could cause injury to MSCs. Thus, we assessed the cytotoxic effects of recipient T cells on ADSCs under various conditions by analyzing ADSC viability. Subsequently, we assessed the cytotoxic effects of recipient T cells on XF-ADSCs, from which factors affecting the cytotoxic action of T cells were removed, by analyzing ADSC death. This study was the first to examine the cytotoxic effects of recipient T cells on allogeneic ADSCs under various conditions. In addition, the effects were evaluated in a human ADSC model to optimize conditions for clinical applications.

ADSCs can be grown in different culture conditions in different laboratories; for example, they can be cultured in medium containing xenogeneic serum for proliferation. Thus, we compared the induction of T cell-mediated cytotoxicity by allogeneic ADSCs previously exposed to xenogeneic serum to that by XF-ADSCs. The autologous serum used was diluted in xeno-free medium to exclude the effects of foreign antigens^37,39,40^. As shown in Figs. 1 and 2, the cell viability of ADSCs previously exposed to xenogeneic serum was decreased to a significantly greater extent by CD8 T cell-mediated cytotoxicity than that of XF-ADSCs. This phenomenon was observed not only in CD8 T cells but also in CD3 T cells as the reaction time increased. The cytotoxic action was induced by granzyme B secreted from alloreactive T cells (Fig. 2c). Our results are supported by previous studies that have raised concerns about the safety of FBS-based culture media because these media induce an immune response^40^. Although FBS and BSA may have different effects on the human immune system, this study has shown that BSA, a major component of FBS, can play an important role in the immune effects. In addition, one thing to consider in Fig. 2d is that the amount of BSA in 10% FBS can be less than the amount of BSA in 10% BSA. This may have affected the IFN-γ production of T cells. These results suggest that culturing allogeneic ADSCs in xeno-free medium containing autologous serum, rather than xenogeneic serum, is the most effective way to grow ADSCs with decreased immunogenicity.

ADSCs have immunosuppressive effects and are expected to be clinically useful therapeutic agents for patients with various immune diseases. We also investigated the cytotoxic effects of T cells on XF-ADSCs pre-exposed to inflammatory conditions and found that the viability of pretreated XF-ADSCs was significantly decreased compared with that of untreated XF-ADSCs 14 days after allogeneic antigen stimulation (Fig. 3a–c). These results are further supported by the fact that pretreated XF-ADSCs induced significantly higher granzyme B secretion from CD8 T cells than untreated XF-ADSCs (Fig. 3d). Collectively, these findings demonstrate that the susceptibility of ADSCs to rejection depends on the inflammatory environment in patients with immune disorders. These results suggest that the greater cytotoxic effect of CD8 T cells on pretreated XF-ADSCs compared to untreated XF-ADSCs may be associated with increased expression of HLA-ABC on these cells due to pretreatment.

This study compared the cytotoxicity of T cells towards allogeneic XF-ADSCs cultured in xeno-free medium immediately after isolation with that towards ADSCs previously exposed to foreign antigens or inflammatory conditions. Interestingly, we found significantly higher granzyme B secretion in CD8 T cells cultured with XF-ADSCs than in control solutions (with CD8 T and Td-PBMC) not containing XF-ADSCs (Fig. 3d). In the antigen recognition pathway analysis of alloreactive T-cell activity, XF-ADSCs induced the activity of alloreactive T cells mainly through direct pathways, suggesting the possibility of allograft rejection through HLA surface antigens (Fig. 4a). Thus, we analyzed the cytotoxic effect of T cells on XF-ADSCs through cell death analysis and found that allogeneic XF-ADSCs displayed significantly more cell death than XF-ADSCs not containing recipient immune cells (Fig. 4b, c). However, XF-ADSCs delayed the cytotoxic action of T cells by approximately 1 week compared to ADSCs pre-exposed to xenogeneic serum or proinflammatory cytokines (Figs. 2–4). These results suggest that although allogeneic XF-ADSCs cultured in autologous serum in a noninflammatory environment may exhibit lower immunogenicity than ADSCs cultured in xenogeneic serum or under inflammatory conditions, they still induce cytotoxicity.

The immune system not only plays an important role in protecting the body from pathogens, but also mediates allogeneic transplant rejection, with CD4 and CD8 T cells recognizing allogeneic HLA-DR and HLA-ABC antigens, respectively. We investigated the causes of recipient CD8 T-cell cytotoxicity towards allogeneic XF-ADSCs using antibodies against HLAs and proinflammatory cytokines. As shown in Fig. 3e, consistent with previous reports, ADSCs expressed HLA-ABC surface antigens even in the absence of stimulation. Furthermore, the antigens were further increased when the cells were exposed to complex mixtures of proinflammatory cytokines, such as those present in autoimmune diseases. These results indicate that the action of alloreactive CD8 T cells against ADSCs may be partially related to the amounts of HLA-ABC expressed. As a result, the cytotoxic action of T cells was markedly decreased in the presence of HLA-blocking antibodies (Fig. 5). Our results demonstrating that HLAs on ADSCs are a major cause of alloreactive CD8 T cell-mediated cytotoxicity are consistent with previous reports that rejection of allogeneic mouse MSCs is attributable to MHC mismatch^16^. In addition, this study showed that CD8 T cells, but not CD4 T cells, are cytotoxic to allogeneic ADSCs and that granzyme B is secreted by CD8 T cells. Taken together, these results indicate that XF-ADSC death can be induced through granzyme B secretion from recipient CD8 T cells that recognize HLA-ABC expressed on allogeneic ADSCs. This suggests that even if HLA-ABC is expressed at low levels on allogeneic ADSCs, it may be a major cause of CD8 T cell-mediated cytotoxicity. In addition, these results suggest that the production of CD8 T cells alloreactive to MSCs might cause second-set rejection after transplantation of other allografts, because the production of CD8 T cells alloreactive against MSCs may lead to the differentiation of memory CD8 T cells. Inhibiting the production of memory CD8 T cells in transplantation remains a great challenge at present. Therefore, we suggest that the transplantation of allogeneic MSCs should be given more attention.

In conclusion, this study showed that allogeneic XF-ADSCs could induce cytotoxicity mediated by recipient CD8 T cells due to HLAs expressed on the ADSC surface. This result also indicated that XF-ADSCs induce T cell-mediated cytotoxicity even though they differ from other allografts due to their immunosuppressive ability. In addition, ADSCs cultured in xenogeneic serum or complex inflammatory milieus can further promote cytotoxicity mediated by recipient CD8 T cells compared to XF-ADSCs. These results suggest that the use of autologous serum is the most effective way to reduce the immunogenicity of allogeneic ADSCs in clinical applications. Ultimately, the use of autologous ADSCs would be a suitable way to address immunogenicity issues, but more attention should be paid to the immunogenicity of allogeneic ADSCs, which can perhaps be addressed through the use of immunosuppressants.
